# Neuroinflammation in the medial prefrontal cortex exerts a crucial role in bone cancer pain

**DOI:** 10.3389/fnmol.2022.1026593

**Published:** 2022-10-25

**Authors:** Xin Li, Wei Wang, Xiaoxuan Zhang, Zhihao Gong, Mi Tian, Yuxin Zhang, Xingji You, Jingxiang Wu

**Affiliations:** ^1^Department of Anesthesiology, Shanghai Chest Hospital, Shanghai Jiao Tong University, Shanghai, China; ^2^School of Medicine, Shanghai University, Shanghai, China; ^3^Department of Critical Care Medicine, Huashan Hospital, Fudan University, Shanghai, China

**Keywords:** transcriptome sequencing, bone cancer pain, medial prefrontal cortex, microglia, immune inflammation, major histocompatibility complex class II

## Abstract

Bone cancer pain (BCP) is one of the most common types of pain in cancer patients which compromises the patient’s functional status, quality of life, and survival. Central hyperalgesia has increasingly been identified as a crucial factor of BCP, especially in the medial prefrontal cortex (mPFC) which is the main cortical area involved in the process of pain and consequent negative emotion. To explore the genetic changes in the mPFC during BCP occurrence and find possible targets for prediction, we performed transcriptome sequencing of mPFC in the BCP rat model and found a total of 147 differentially expressed mRNAs (DEmRNAs). A protein-protein interaction (PPI) network revealed that the DEmRNAs mainly participate in the inflammatory response. Meanwhile, microglia and astrocytes were activated in the mPFC of BCP rats, further confirming the presence of neuroinflammation. In addition, Gene Ontology (GO) analysis showed that DEmRNAs in the mPFC are mainly involved in antigen processing, presentation of peptide antigen, and immune response, occurring in the MHC protein complex. Besides, the Kyoto Encyclopedia of Genes and Genomes (KEGG) analysis revealed that DEmRNAs are mainly enriched in the pathways of phagosome, staphylococcus aureus infection, and antigen processing, in which MHCII participate. Furthermore, immunostaining showed that MHCII is mainly located in the microglia. Microglia are believed to be involved in antigen processing, a key cause of BCP. *In vivo*, minocycline (MC) treatment inhibits the activation of microglia and reduces the expression of MHCII and proinflammatory cytokines, thereby alleviating BCP and pain-related anxiety. Taken together, our study identified differentially expressed genes in the BCP process and demonstrated that the activation of microglia participates in the inflammatory response and antigen process, which may contribute to BCP.

## Introduction

The incidence of cancer worldwide has increased steadily in the past 10 years (Miller et al., 2020), advanced cancer metastasis to bone is one of the most common complications (Meuser et al., 2001; Clézardin et al., 2021), inflicting patients with severe and chronic pain. Unfortunately, current pain management interventions are still insufficient (Kapoor et al., 2021). Several causes of bone cancer pain (BCP) have been proposed, including structural reorganization of sensory and sympathetic nerve fibers in the bone, combined with the cellular and neurochemical reorganization in the spinal cord and brain (Mantyh, 2013; Zaja̧czkowska et al., 2019). More and more evidence has been accumulated to support the role of central nociceptive hypersensitivity in BCP (Hou et al., 2020; Wu et al., 2021).

The medial prefrontal cortex (mPFC) is a central mediator in the pain pathway, which is crucial as an integrator of ascending sensory information, cognitive and emotional responses, and descending inhibitory control (Ong et al., 2019; Kummer et al., 2020). Changes in gene expression in the PFC play a role in acute and chronic pain development and maintenance (Yan et al., 2019; Cai et al., 2020; Qian et al., 2020; Shiers and Price, 2020; Topham et al., 2020). During chronic pain states, it has been reported that mPFC undergoes structural and functional plasticity (Obermann et al., 2013; Liu et al., 2018; Sang et al., 2018; Huang et al., 2020). Transcriptome analysis of the mPFC using ribonucleic acid (RNA) sequencing and further RT-qPCR has also shown that rodents with neuropathic pain displayed an increase in genes including NMDA receptor, BDNF, and κ-opioid receptor (Alvarado et al., 2013; Palmisano et al., 2019). Most recently, DNA methylation in the PFC was found to be highly correlated with both pain and comorbid behaviors in a mouse model of neuropathic pain (Tajerian et al., 2013; Topham et al., 2020). In addition, mPFC microglia have been shown to cause pain in the model of the peripheral nerve injury (Fiore and Austin, 2019). Therapeutic mechanisms targeting mPFC may have efficacy across various pain disorders and simultaneously address associated cognitive and emotional comorbidities (Nashed et al., 2015). However, when BCP occurs, the expression profile and specific BCP regulation mechanisms of mRNA in the prefrontal cortex are largely unknown.

To detect transcriptomic changes in the mPFC of the BCP process and explore the potential therapeutic targets, we established the widely used BCP rat model and performed transcriptome sequencing to explore expression profile changes of mRNAs, and functional analysis of differential genes to provide new insights on the mechanism of BCP. Differentially expressed mRNAs (DEmRNAs) were subjected to Gene Ontology (GO) and Kyoto Encyclopedia of Genes and Genomes (KEGG) pathway analyses to evaluate their functions and possible mechanisms. Our study may provide new insights into understanding the central mechanisms of BCP and help to find promising genes or signaling pathways that could be manipulated to treat BCP disorders.

## Materials and methods

### Bone cancer pain animal model

Female Sprague-Dawley (200–250 g) and Wistar rats (60–70 g) were purchased from B&K Universal Group Limited (Shanghai, China) and housed in cages at a temperature of 24 ± 1°C under 12/12 h light-dark cycles with free access to food and water. All animal handling and surgical procedures were carried out in accordance with the guidelines of the International Association for the Study of Pain, approved by the Animal Care and Use Committee of Shanghai Chest Hospital, Shanghai Jiao Tong University. In the study, Sprague-Dawley rats weighing 200–250 g were randomly divided into three groups: Sham group, BCP + normal saline group (BCP group), and BCP + 20 mg/kg MC group (BCP + MC group).

BCP was induced in rats as previously described (Brigatte et al., 2007). Briefly, female Wistar rats received an intraperitoneal inoculation of Walker 256 mammary gland carcinoma cells (purchased from ATCC). After 1 week, cells in the ascites were collected and resuspended in saline to a final concentration of 2 × 10^7^ cells/ml. Next, female Sprague Dawley rats were anesthetized with pentobarbital (50 mg/kg, i.p.), and a 23-gauge needle was inserted into the intramedullary canal of the right tibia at one-third of the length from the lower end, to create a cavity for the injection of saline (10 μl, Sham) or Walker 256 cell suspension (10 μl, containing 2 × 10^5^ cells in total, BCP). The cavity was sealed with bone wax and the incision was closed with stitches. Upon waking from anesthesia, the animals were returned to their home cages.

### Pain behavioral tests

Mechanical allodynia was evaluated by measuring the paw withdrawal thresholds (PWT) in response to a series of calibrated von Frey filaments (Remeniuk et al., 2015). In brief, rats were transferred to a chamber with a metal mesh floor and allowed to acclimatize for 30 min. Then, von Frey filaments were applied to the plantar surface of the right hind paw in ascending order (1, 1.4, 2, 4, 6, 8, 10, and 15 g). Abrupt paw withdrawal, licking, and shaking are defined as positive responses. Once a positive response was observed, the rat was allowed to rest for 5 min. PWT was defined as the lowest force that elicited a positive response and averaged across three repeats of measurement.

Movement-evoked pain was assessed with the limb use score (LUS) (Remeniuk et al., 2015). Rats were permitted to move spontaneously on a smooth plastic table (50 × 50 cm). The limb use during spontaneous ambulation was scored on a scale of 4–0 (4: normal use; 3: slightly limping; 2: limping; 1: no use of the limbs, partial; 0: no use of the limbs, complete).

Microglia-activated inhibitor MC was purchased from MedChemExpress (Monmouth Junction, NJ, USA), dissolved in saline (20 mg/ml, stored at −20°C for use), and administrated every other day from 1 day after surgery (D1, 20 mg/kg, i.p.). All behavioral tests were performed by an investigator who was blinded to the experimental design.

### Elevated plus maze test

Anxiety-like behavior was measured using the elevated plus-maze (Shanghai Jiliang Software Technology Co., Ltd.). It consisted of two opposing open arms (50 × 10 × 0.5 cm), two opposing closed arms (50 × 10 × 40 cm), and a central open platform (10 × 10 cm). Each rat was placed on the central platform facing one of the open arms and allowed to explore the apparatus for 5 min. We analyzed the time spent in the open arms and total numbers of entries by JLBehv-EPMG software, based on previous literature (Masukawa et al., 2020).

### Tissue collection and ribonucleic acid extraction

On day 17, rats were deeply anesthetized with sodium pentobarbital (40 mg/kg, i.p.) and perfused through the ascending aorta with 0.9% saline (4°C). After the perfusion, the medial prefrontal central (mPFC) was collected. Total RNA was extracted from the Sham and BCP group tissues using Trizol reagent (Invitrogen, Carlsbad, CA, USA), following the manufacturer’s instructions. For tissue samples, about 60 mg of liquid nitrogen was ground into powder and the powder samples were transferred into 1.5 ml Trizol reagent. The mixture was centrifuged at 12,000 × *g* for 5 min at 4°C. The supernatant was transferred to a new 2.0-ml tube with 0.3 ml of chloroform/isoamyl alcohol (24:1) per 1.5 ml of Trizol reagent. After the mixture was centrifuged at 12,000 × *g* for 10 min at 4°C, the aqueous phase was transferred to a new 1.5 ml tube which was added with an equal volume of supernatant of isopropyl alcohol. The mixture was centrifuged at 12,000 × *g* for 20 min at 4°C and then the supernatant was removed. After being washed with 1 ml 75% ethanol, the RNA pellet was air-dried in the biosafety cabinet and then dissolved by adding 25 ∼ 100 μl DEPC-treated water. Subsequently, total RNA was qualified and quantified using a Nano Drop and Agilent 2100 Bioanalyzer (Yin et al., 2021) (Thermo Fisher Scientific, MA, USA).

### RNA-seq library establishment and RNA-seq

Approximately 1 μg total RNA per sample was treated with Ribo-Zero^®^ Magnetic Kit (Epicenter) to deplete rRNA. First-strand cDNA was generated using random primers reverse transcription, followed by second-strand cDNA synthesis. The synthesized cDNA was subjected to end-repair and then was 3′ adenylated. Adapters were ligated to the ends of these 3′ adenylated cDNA fragments. Several rounds of PCR amplification with PCR Primer Cocktail and PCR Master Mix are performed to enrich the cDNA fragments. Then the PCR products are purified with Ampure XP Beads. The final library was quality and quantitated in two methods: checking the distribution of the fragment size using the Agilent 2100 Bioanalyzer and quantifying the library using real-time quantitative PCR (qPCR) (TaqMan Probe). The qualified libraries were sequenced pair end on the Hiseq 4000 platform (Fang et al., 2018) (BGI-Shenzhen, China).

### Bioinformatics analysis

Primary sequencing data produced by RNA-Seq (raw reads) were subjected to quality control (QC). The sequencing data was filtered with SOAPnuke (v1.5.2) by removing reads containing sequencing adapter; removing reads whose low-quality base ratio (base quality less than or equal to 5) is more than 20%, and removing reads whose unknown base (“N” base) ratio is more than 5%; afterward, clean reads were obtained and stored in FASTQ format. The clean reads were mapped to the reference genome using HISAT2 (v2.0.4). After that, Ericscript (v0.5.5) and rMATS (V3.2.5) were used to detect fusion genes and differential splicing genes (DSGs), respectively. Bowtie2 (v2.2.5) was applied to align the clean reads to the gene set, a database built by BGI (Beijing Genomic Institute in Shenzhen), in which known and novel, coding and non-coding transcripts were included, then the expression level of the gene was calculated by RSEM (v1.2.12). The heatmap was drawn by pheatmap (v1.0.8) according to the gene expression in different samples. Essentially, differential expression analysis was performed using the DESeq2(v1.4.5) with *q*-value ≤ 0.05.

### Cluster analysis and screening of differentially expressed genes

Distances of expressed genes were calculated using the Euclidean method. The sum of the squared deviations algorithm was used to calculate distance. The cluster analysis and heat map visualization of gene expression patterns were performed using the “pheatmap” package in the R software of Bioconductor. DEmRNAs with statistical significance were identified through Scatter Plot filtering as we reported before. The threshold required for the results to be considered significant was as follows: *q*-value ≤ 0.01 and the absolute value of | log2 (fold change) | ≥ 0.58 as in our previous study. To take an insight into the change of phenotype, GO and KEGG enrichment analysis of annotated different expression genes was performed by bioinformatics.^1^

### Western blot

Animals were deeply anesthetized with sodium pentobarbital (50 mg/kg, i.p.) and rapidly sacrificed by cervical dislocation. The mPFC was homogenized in RIPA buffer containing 1 mM PMSF (Beyotime) and centrifuged at 13,000 × g for 30 min. The supernatant was collected, and the total protein concentration was titrated using a bicinchoninic acid kit. An equivalent amount of protein (50 μg) was fractionated on 10% polyacrylamide gel. Proteins were then transferred to nitrocellulose membranes (Millipore) at 300 mA for 90 min. Membranes were blocked with 5% BSA in Tris-buffered saline (TBS, 50 mM Tris-HCl, 150 mM NaCl, pH 7.5) for 2 h at room temperature and incubated overnight at 4°C with primary antibodies (anti-CD74 at 1:500, sc-6262, SANTA CRUZ; anti-CTSS at 1:1,000, ab92780, Abcam; anti-CIITA at 1:1,000, sc-13556, SANTA CEUZ; anti-Vinculin at 1:1,000, A2752, ABclonal; anti-MHCII at 1:1,000, sc06-78, invitrogen) in TBS containing 5% BSA. Following three washes with TBS, membranes were incubated with horseradish peroxidase-conjugated secondary antibodies (goat anti-rabbit at 1:2,000, ab205718, Abcam; goat anti-mouse at 1:2,000, ab205719, Abcam) in TBS containing 5% BSA for 2 h at room temperature. Immunoreactive proteins were visualized using the enhanced chemiluminescence western blotting detection system (Santa Cruz). The membranes were analyzed with a computerized image analysis system (ChemiDoc XRS1, Bio-Rad, Hercules, CA), and the intensity of the protein bands was quantified with Image Lab software (ImageJ). All protein expression was normalized to Vinculin.

### Bone histomorphometric analysis

The ipsilateral tibia of BCP rats was fixed in 4% paraformaldehyde for 48 h, followed by decalcification in 10% EDTA for 3–4 weeks, and 6-μm sections were prepared on a rotating microtome. Paraffin-embedded sections were deparaffinized in xylene, rehydrated, and stained with hematoxylin-eosin (H&E; Sigma, St Louis, MO, USA) according to the manufacturer’s protocol (Wang et al., 2019).

### Enzyme-linked immunosorbent assay

ELISA was used to determine the inflammatory cytokines (IL-1β, TNF-α, and IL-18) in the mPFC. The samples were weighed and then homogenized completely in phosphate-buffered solution (PBS). After centrifugation (1,000 × g, 10 min) of the homogenates at 4°C, supernatants were collected and stored at -80°C. The concentrations of TNF-α (ml002859-C), IL-1β (ml037361-C), and IL-18 (ml002816-C) were detected following the instruction of ELISA kits (Shanghai Enzyme-linked Biotechnology Co., Ltd.).

### Real-time quantitative polymerase chain reaction

Total RNA was extracted from mPFC using a total RNA Kit (Vazyme Biotech Co., Ltd., China), and complementary DNA (cDNA) was generated using a cDNA Synthesis Kit (DBI^®^ Bioscience Co., Ltd., Germany), according to instructions provided by the manufacturers. RT-qPCR was carried out using an SYBR Green assay (DBI^®^ Bioscience) on a StepOnePlus Real−Time PCR System (Applied Biosystems^®^, Thermo Fisher Scientific, Waltham, MA, USA), and the relative gene expression was determined using the comparative Ct (ΔΔCt) method. GAPDH was used as a housekeeping gene. The primer sequences are as Supplementary Table 1.

### Immunofluorescence

Animals were deeply anesthetized with sodium pentobarbital (50 mg/kg, i.p.) and underwent sternotomy, followed by intracardiac perfusion with 200 ml saline and 200 ml 4% ice-cold paraformaldehyde in 0.1 M phosphate-buffered saline. The brain was removed, postfixed in 4% paraformaldehyde for 4 h, and subsequently allowed to equilibrate in 30% sucrose in phosphate-buffered saline overnight at 4°C. Brain samples were embedded in Tissue-Tek O.C.T., cut at a thickness of 20 μM using a cryostat (Leica CM1850-1-1), and mounted on slides. All Brain samples were thoroughly rinsed in PBS to remove any residual OCT. The samples were then blocked in 0.3% Triton-X 100 and 5% normal donkey serum (NDS) in PBS for 1 h, and incubated overnight with anti-MHCII (1:50, MCA46GA, BIO-RAD), anti-IBA-1 (1:100, ab178847, Abcam), anti-GFAP (1:100, ab254082, Abcam), anti-NEUN (1:100, ab177487, Abcam). On the next day, the samples were rinsed in PBS and incubated for 1 h in PBS containing appropriate secondary antibody (1:500) from Jackson ImmunoResearch (catalog nos. 711-545-152 or 715-585-150) or Cell Signaling Technology (catalog nos. 8,889 or 4,408). The samples were then rinsed in PBS and coverslipped with Antifade Mounting Medium with DAPI (Beyotime, Nanjing, China). The imaging and subsequent analysis were performed using a Confocal Microscope (TCS SP8, Leica Microsystems, Mannheim, Germany).

### Statistics

Data are reported as the mean ± standard deviation. The data were analyzed using GraphPad Prism 8.0 for Windows (San Diego, CA, USA). Results from behavioral testing were analyzed using ANOVA for repeated measures. For all other measures between the 2 groups, the Student’s *t*-test was used for the comparisons of variables with normal distribution from independent samples. For comparisons among > 2 groups, a one-way ANOVA with Turkey’s multiple comparisons test was applied. Two-way ANOVA followed by Turkey’s multiple comparisons test with repeated measurements was used to determine the interaction between time and two groups. Fisher’s least significant difference test for pairwise comparisons, all statistical tests were two-sided, and *p* < 0.05 was considered statistically significant.

## Results

### Rats developed mechanical hypersensitivity and osteolytic lesions after tumor cells inoculation

**FIGURE 1:**
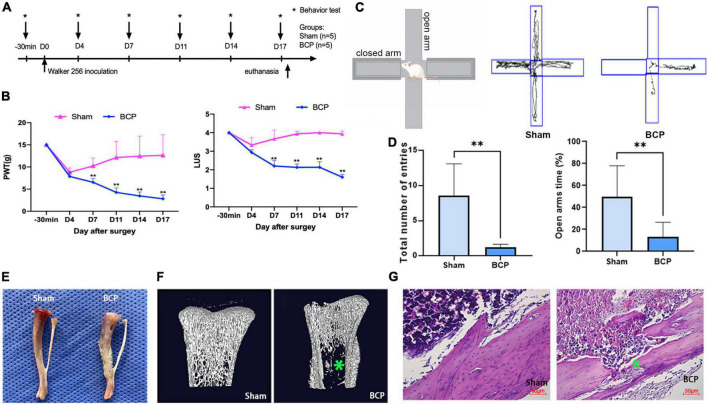
Induction of bone cancer pain (BCP) in rats. **(A)** Experimental timeline showing the dates of surgery (D0) and behavioral tests (−30 min, D4, D7, D11, D14, and D17). On D0, saline (Sham) or Walker 256 mammary gland carcinoma cells (BCP) were injected into the tibia in rats and pain sensation was assessed before and after through behavioral tests. **(B)** Induction of significant hyperalgesia in the tumor-bearing hind paw, as assessed through the paw withdrawal threshold (PWT, **Left**) and the limb use score (LUS, **Right**). **(C,D)** Track and result in an analysis of cross maze. A total number of entries and times in open arms were used to measure the degree of walking ability and pain-related anxiety. Compared with the Sham group, the walking ability of BCP group was decreased and exerted pain-related anxiety. **(E)** Photograph of the tibia from a BCP rat on D17 shows obvious tumor growth and bone mass destruction **(Right)** compared to the one from a Sham rat **(Left)**. **(F)** Representative micro-CT images showing the bone microstructure of two tibias from a Sham rat **(Left)** and a BCP rat **(Right)** on D17. Note the reduction in medullary bone and the increase in lesions in cortical bone with the tibia from the BCP rat. **(G)** Hematoxylin and eosin-stained histological sections show obvious medullary bone loss and bone destruction in the tibia of a BCP rat on D17 **(Right)** when compared to the one from a Sham rat **(Left)**. Data are expressed as mean ± SD and statistically analyzed by Two-way ANOVA followed by Turkey’s multiple comparisons test with repeated measurements, the behavior test has been repeated 3 times, and *p* < 0.05 was considered statistically significant. *n* = 5 per group. *p < 0.05, ^**^p < 0.01 vs. Sham groups.

To induce BCP in rats, we injected Walker 256 mammary gland carcinoma cells into the intramedullary canal of the right tibia. Behavioral tests were performed 30 min before the operation and on D4, D7, D11, D14, and D17 (Figure 1A). BCP rats exhibited a significant decrease in PWT and LUS when compared to the Sham rats (Figure 1B), consistent with previously reported values (Remeniuk et al., 2015). Meanwhile, the elevated plus-maze experiment carried out on D17 showed the total number of entries and open arms time (%) of BCP rats were reduced (Figures 1C,D), indicating that they suffered from pain-related anxiety and impaired walking ability (Blaszczyk et al., 2018). Next, a pathological examination was conducted on D17 after the animals were sacrificed. We found that in gross images the surface of the tibia of Sham rats was smooth while that of BCP rats was swollen and uneven (Figure 1E). Furthermore, we found bone destruction in BCP rats, in contrast to uniform bone quality in Sham rats with tibia micro-CT (Figure 1F). Lastly, we performed hematoxylin and eosin staining of the tibia and found tumor cell proliferation and invasion of the bone cortex in BCP rats, but not in Sham rats (Figure 1G). Taken together, these behavioral and pathological results strongly suggested that BCP was successfully induced in rats.

### Transcriptomic high-throughput sequencing revealed changes in gene expression profiles

**FIGURE 2:**
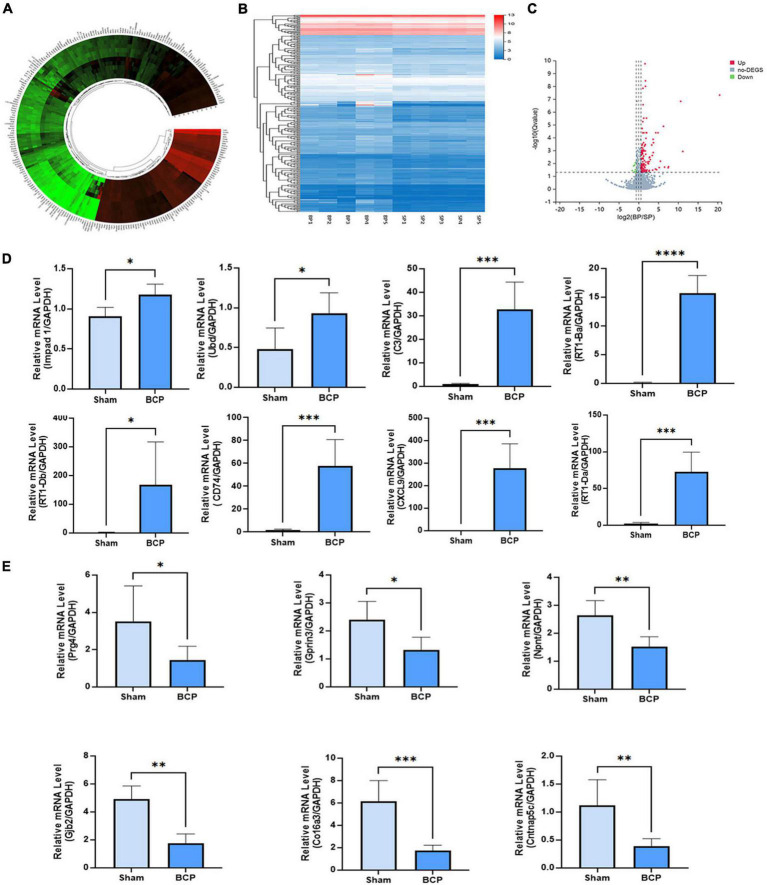
DEmRNAs in the mPFC were found between Sham and BCP rats at D17. **(A)** The gene map of the different expression mRNAs (DEmRNAs) in the mPFC between Sham and BCP rats. **(B)** The heatmap of the DEmRNAs in the mPFC between Sham and BCP rats. **(C)** The volcano plot of DEmRNAs in the mPFC between Sham and BCP rats. **(D,E)** The expression level of up-regulated mRNAs **(D)** and downregulated mRNAs **(E)**, with gene names on each bar graph. Data are expressed as mean ± SD and statistically analyzed by Student’s *t*-test. *n* = 5 per group. *p < 0.05, ^**^p < 0.01, ^***^p < 0.001, ^****^p < 0.0001 vs. Sham group.

The count and fold change of DEmRNAs were visualized by a gene map (Figure 2A). The expression of ten mRNAs was changed more than 20 times in the mPFC of BCP. Hierarchical clustering of the expression of mRNA showed obvious discrimination between BCP and Sham rats (Figure 2B). The results were visualized through a volcano plot (Figure 2C). These results indicated that tumor inoculation in the tibia shifted expression profiles of mRNA in mPFC, and 147 DEmRNAs (136 upregulated and 11 downregulated) were found in the transcriptomic sequencing, which may account for the development of hyperalgesia in BCP rats.

To validate the reliability of sequencing quantification, 18 DEmRNAs (10 upregulated and 8 downregulated) were selected to undergo RT-qPCR on another batch of samples (*n* = 5 per group), independent of the ones used for RNA-seq. As shown in Figure 2D, RT-qPCR quantification of top DEmRNA was consistent with RNA sequencing; Impad1, Ubd, RT1-Ba, RT1-Da, RT1-Db, CXCL9, Cd74, C3, and CXCL9, were all significantly upregulated (Figure 2D), and Prg4, Gprin3, Npnt, Gjb2, Co16a3, and Cntnap5c were downregulated (Figure 2E).

### Protein-protein interaction network of differentially expressed mRNAs

**FIGURE 3:**
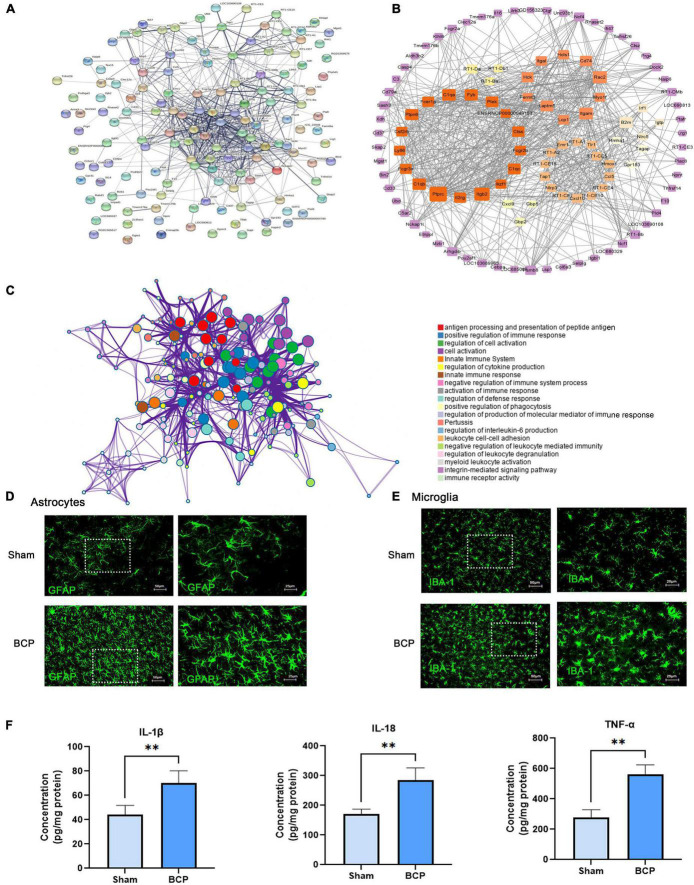
Immune inflammation exerts a crucial role in bone cancer pain at D17. **(A)** PPI network constructed by the 147 DEmRNA-coding protein. **(B)** The node’s color scale indicates connectivity with other nodes, and the edge’s thickness indicates the interaction’s combined score. The clusters were the top seven subnetworks calculated by MCODE. **(C)** A functional enrichment network is constructed by nodes representing terms. **(D,E)** Astrocyte and microglia activated in the mPFC of BCP rats. Representative immunofluorescence images showing the morphologies of the astrocyte labeled with GFAP **(D)**, microglia labeled with Iba1 **(E)** in the mPFC. **(F)** The pro-inflammatory cytokines upregulated in the mPFC of BCP rats. Data are expressed as mean ± SD and statistically analyzed by Student’s *t*-test. *n* = 5 per group. **p < 0.01, vs. Sham groups.

For the 147 DEmRNAs, we obtained the PPI network containing 112 nodes and 674 edges. The network was set to the default cutoff (interaction score > 0.4) in the STRING online database (Figure 3A). It showed extensive interaction among them and 7 subnetworks were clustered, which may represent more compact functional blocks (Figure 3B). Moreover, functional analysis of these genes showed that they were mainly involved in immunological processes/pathways, indicating they were crucial to the pathogenesis of BCP (Figure 3C). In the central system, astrocytes and microglia may release various inflammatory mediators that stimulate nociceptive neurons and reinforce glial activation, thereby promoting neuronal sensitization and behavioral hypersensitivity observed in neuropathic pain (Blaszczyk et al., 2018). In our study, immunostaining showed that morphology and number of astrocytes and microglia are changed in the mPFC of BCP rats (Figures 3D,E). Furthermore, ELISA analysis showed that the pro-inflammatory cytokines level of IL-1β, IL-18, and TNF-α were upregulated in the mPFC of BCP rats (Figure 3F), suggesting the inflammatory response.

### Gene ontology analysis and Kyoto Encyclopedia of Genes and Genomes analysis of differentially expressed mRNAs

**FIGURE 4:**
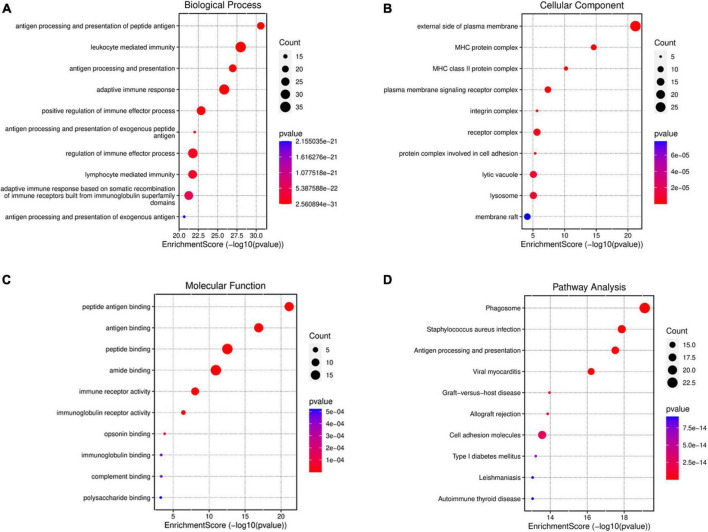
GO and KEGG enrichment analysis for DEmRNAsof mPFC in the progress of BCP. GO enrichment of DEmRNAs shows the top 10 terms (according to *p*-value) in three types of GO terms, biological process **(A)**, cellular component **(B)**, and molecular function **(C)**. **(D)** KEGG enrichment analysis shows the top 10 pathways in the progress of BCP. The size of the bubble indicates the number of enriched DEmRNAs, the color indicates the *p*-value and the position indicates the rich factor.

In order to further explore the function of DEmRNAs, GO enrichment analysis was conducted on 147 DEmRNAs, and results showed that the DEmRNAs were mainly involved in the following biological process (BP) terms: antigen processing and presentation of peptide antigen, leukocyte mediated immunity, antigen processing and presentation, and so forth (Figure 4A). Most DEmRNAs were enriched in the following cellular component (CC) terms: external side of the plasma membrane, MHC protein complex, MHC class II protein complex, and so forth (Figure 4B). Meanwhile, the following molecular function (MF) terms were enriched including peptide antigen binding, and immunity receptor activity (Figure 4C).

Similarly, KEGG analysis of these dysregulated genes showed that, neglecting unrelated pathways, DEmRNAs were primarily enriched in antigen processing and presentation, phagosome, staphylococcus aureus infection, etc. (Figure 4D). These results also indicated the involvement of extra-/intra-cellular pathways in inflammatory and immunologic processes.

### MHCII in microglia of medial prefrontal cortex may participate in the process of bone cancer pain

**FIGURE 5:**
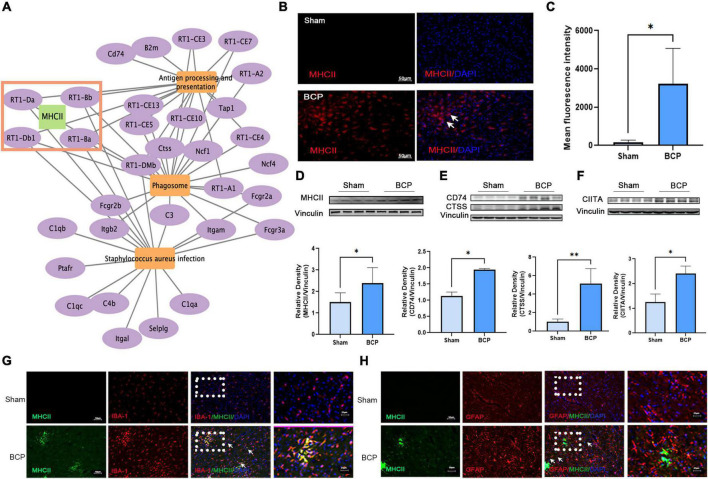
Increased expression of MHCII in the medial prefrontal cortex (mPFC) in rats with BCP at D17. **(A)** The interaction of the top 3 pathways and genes showed that MHCII may exert a crucial role. **(B,C)** Immunofluorescent showed the increased expression of MHCII. **(D–F)** Western blot also revealed that MHCII was upregulated **(D)**. Western blot revealed that the CD74 and CTSS upstream of MHCII and CIITA downstream of MHCII were upregulated in the mPFC of BCP rats **(E)**. *p < 0.05, **p < 0.01 vs. Sham group. **(G,H)** Double immunofluorescent staining of MHCII and cell type-specific markers revealed that the increased expression of MHCII in the mPFC of rats with BCP occurred mainly in microglia **(G)**, but not in astrocytes **(H)**.

We aimed to explore the underlying neuroimmune mechanism of BCP and find several valuable pain biomarkers. It was found that MHCII may be involved in the top three pathways: antigen processing and presentation, phagosomes, and staphylococcus aureus infection (Figure 5A). We investigated the expression of MHCII in the mPFC after BCP. RT1-Da and RT1-Bb, two subunits of MHCII, were strongly upregulated at the mRNA level in BCP rats when compared to Sham rats (Figure 2D and Supplementary Figure 1). Consistently, both western blot and immunostaining confirmed increased expression of MHCII in the mPFC of BCP rats at the protein level (Figures 5B–D). Remarkably, the expression of CD74 and CTSS, two binding partners of MHCII in downstream antigen presentation, and the class II trans-activator (CIITA), a key transcription factor for MHCII, were all significantly increased in BCP rats when compared to Sham rats (*p* < 0.05, Figures 5E,F). Therefore, MHCII may be involved in the process of BCP. In addition, double immunostaining revealed that MHCII was localized mainly in microglia, not in astrocytes (Figures 5G,H). Microglial cells, rather than astrocytes, are considered to be the main antigen-presenting cells in the central nervous system (Almolda et al., 2011; Dasgupta and Dasgupta, 2017), suggesting that MHCII in the mPFC may be a biomarker of activated microglia which participate in the antigen process to contribute to BCP.

**FIGURE 6:**
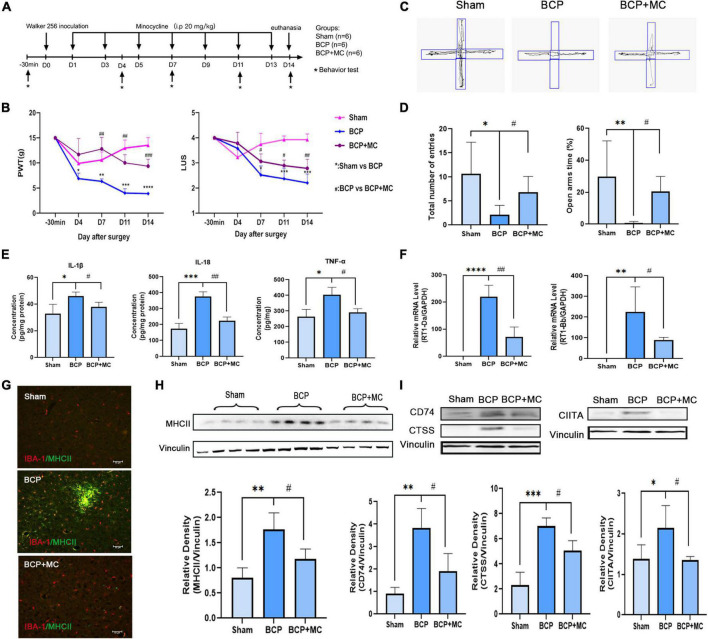
The inhibition of activation of microglia administrated relieved hyperalgesia and pain-related anxiety at D14. **(A)** Experimental timeline showing injection of minocycline (20 mg/kg, i.p.) every other day from D1. **(B)** Both PWT (left) and LUS (right) of BCP rats were elevated by minocycline **(C,D)**. Total number of entries and times in open arms reduced in the BCP rats reversed after the treatment of minocycline. **(E)** The increased expression of proinflammatory cytokines was downregulated in the BCP + MC group. **(F–I)** The upregulated expression of MHCII **(F–H)** and the related protein CIITA, CTSS, and CD74 were both decreased in the BCP + MC group **(I)**. *p < 0.05, ^**^p < 0.01, ^***^p < 0.001, ^****^p < 0.0001 vs. Sham group; ^#^*p* < 0.05, ^##^*p* < 0.01, ^###^*p* < 0.001 vs. BCP group. Error bars are represented as mean ± SD, one-way analysis of variance (ANOVA) followed by Tukey’s multiple comparison test, *n* = 4 per group.

In order to further investigate whether activation of microglia in mPFC plays a crucial role in the process of BCP, minocycline (MC) was administered in BCP rats to inhibit microglia activation (20 mg/kg, every other day from D1, intraperitoneally) (Figure 6A). Rats were randomly divided into three groups; Sham, BCP (+ saline), and BCP + MC. The reduction of PWT and LUS in BCP rats can be reversed by MC treatment (Figure 6B). Meanwhile, the elevated plus-maze experiment carried out at D14 showed that the MC treatment increased the total number of entries and open arms time (%) (Figures 6C,D), indicating improvement of walking ability and relief of pain-related anxiety. In addition, the upregulation of pro-inflammatory cytokines of BCP was reversed in the BCP + MC group (Figure 6E), confirming the inhibition of microglia activation may relieve the symptom of BCP. To detect the expression of antigen process, RT-PCR, western blot, and immunofluorescent staining showed that the upregulation of MHCII in the mPFC microglia was decreased (Figures 6F–I) and the upregulation of its downstream binding partners (CD74 and CTSS) and key transcription factor (CIITA) (Figures 6H,I) were also reversed after the treatment of MC. It indicates that MHCII and its related pathway may be decreased after the inhibition of microglia activation. Thus, we proposed that MHCII in the mPFC may be a biomarker for microglia activation to participate in antigen processing and further promote BCP.

## Discussion

In this study, the whole genome of mPFC from rats with BCP was sequenced for the first time, revealing the key genes, which was a highlight. Firstly, the transcription sequence showed that there are 147 DEmRNAs in the mPFC of the BCP rat. Secondly, KEGG and GO enrichment indicated that the DEmRNAs are mainly involved in immune inflammation. Thirdly, we displayed that MHCII, which is the key gene in the enrichment, was increased in the microglia of BCP rats’ mPFC. Lastly, we confirmed that the pain and pain-related anxiety were relieved by administrating MC to inhibit microglia activation, accompanied by the reversal of the upregulation of MHCII, indicating that MHCII related to microglia activation plays a crucial role in the mPFC of BCP.

A wealth of evidence has suggested that BCP is a complex clinical syndrome, that shares several features with inflammatory and neuropathic pain (Falk and Dickenson, 2014) and which often comes with concurrent depression, cognitive impairment, and other diseases (Feller et al., 2019; Liu et al., 2021). The mPFC is a main cortical area involved in processing both pain and consequent negative emotion (Salomons et al., 2015; Mecca et al., 2021), notably, it has been reported that dysfunction of mPFC neurons may be implicated in neuropathic pain-related anxiety (Du et al., 2022). Most, recently, Dai et al. (2022) performed a gene set enrichment analysis in the mPFC of neuropathic pain, and they found that there are 49 different expression genes. To further explore the potential targets in the mPFC of BCP, we established a comprehensive transcription sequence. The analysis showed that there were 147 DEmRNAs. PPI network analysis showed that DEmRNAs in mPFC were mainly involved in immune inflammation. Meanwhile, in the mPFC of BCP rats, microglia and astrocytes were activated, and pro-inflammatory cytokines were released, indicating an immune inflammation response. In addition, accumulating evidence suggests that in the central nervous system non-neuronal cells interact with nociceptive neurons by secreting neuroactive signaling molecules that modulate pain (Ji et al., 2016; Totsch and Sorge, 2017).

In the analysis of neuropathic pain, the most significantly regulated gene sets included oligodendrocyte differentiation, semaphoring plexin signaling pathway, negative regulation of gliogenesis, neuron fate commitment, intrinsic component of external side of plasma membrane, and germ cell nucleus (Dai et al., 2022). While, in our study, different from neuropathic pain, we found that the BP, CC, and MF was related to the MHC complex and antigen process in the mPFC of BCP rats. As previous research reported that infiltrated CD4^+^T lymphocytes might interact with MHCII-presented antigen in the spinal cord and contribute to BCP (Song et al., 2017). As such, our study proposed that MHCII in the mPFC may exert a crucial role in the process of BCP. Besides, KEGG enrichment also displayed that 32 genes were involved in the top three pathways: Phagosome, Staphylococcus aureus infection, Antigen processing, and presentation. Further, MHCII was involved in all three pathways, indicating MHCII in the mPFC plays an important role in the occurrence of BCP.

In line with the literature, MHCII has been shown to participate in the occurrence of different chronic pain (Dominguez et al., 2008a,b; Liu et al., 2012; Hartlehnert et al., 2017; Dorsey et al., 2019). In the present study, the expression of MHCII was upregulated in the mPFC of BCP rats, confirming the key role of MHCII. To further investigate the function of MHCII in the process of BCP, we found that MHCII was located in the activated microglia which is the main antigen-presenting cells population in the central nervous system. Although some studies have shown that activated microglia in the spinal cord and hippocampus may modulate BCP in rats’ model (Dai et al., 2019; Diaz-delCastillo et al., 2020), there are no typical markers indicating the types of microglia involved in pain. In our study, we found that inhibition of the activation of microglia by MC could relieve BCP as well as pain-related anxiety. It is worth noting that the upregulated expression of MHCII could be reversed by the treatment of MC, as such, we speculated that microglia co-labeled with MHCII in the mPFC may participate in BCP. Since MC was administered systemically, its analgesic effect may partly due to inhibit microglia and MHCII in other areas of the nervous system, such as spinal cord. However, in this study, we found that MC inhibited microglia and MHCII activation in mPFC, suggesting that at least mPFC play a role in analgesia. To further investigate this mechanism, our future study may focus on mPFC in BCP rats.

In summary, by high-throughput sequencing of the transcriptome, we found 147 DEmRNAs (136 upregulated and 11 downregulated) in the mPFC of BCP rats established by implantation of Walker 256 carcinoma cells into the right tibia. MHCII exerts a crucial role in the transcription analysis and our research supposed that MHCII in the mPFC may be a biomarker for microglia activation to participate in antigen processing and further promote BCP. These findings shed new light on the mechanisms of BCP and open a new venue for designing treatment plans to alleviate BCP in patients with advanced cancer, and additional tests are necessary to provide a more comprehensive picture.

## Data availability statement

The original contributions presented in this study are publicly available. This data can be found here: https://www.ncbi.nlm.nih.gov/, PRJNA876307.

## Ethics statement

This animal study was reviewed and approved by the Animal Care and Use Committee of Shanghai Chest Hospital, Shanghai Jiao Tong University [permission no. KS (Y)20170] and carried out in accordance with the guidelines of the International Association for the Study of Pain.

## Author contributions

JW and XY designed the study. XL, WW, XZ, ZG, MT, and YZ performed the experiments. ZG, XZ, MT, and XY analyzed the data. XL and WW wrote the manuscript. All authors reviewed and approved the manuscript.

## Footnotes

1
www.bioinformatics.com.cn

## Supplementary material

Supplementary Figure 1 The expression level of upregulated mRNAs (RT1-Bb and CTSS) in the mPFC of BCP rats. Data are expressed as mean ± SD and statistically analyzed by Student’s *T*-test. *n* = 5 per group. **p* < 0.05, ***p* < 0.01 vs. Sham group.

Supplementary Table 1 The primer sequences are listed in the Supplementary Table 1.

Supplementary Table 2 A total of 20,258 genes were detected in the mPFC between Sham and BCP rats, each group with *n* = 5.

Supplementary Table 3 The count and fold change of the 147 DEmRNAs in the mPFC between Sham and BCP rats.

Supplementary Table 4 The details of the interactions between the 147 DEmRNA-coding proteins.
