# Supplementary figures and images for: Neuroinflammation in the medial prefrontal cortex exerts a crucial role in bone cancer pain
Source: Front Mol Neurosci. 2022 Oct 25;15:1026593. doi: 10.3389/fnmol.2022.1026593 (PMC9642970; doi:10.3389/fnmol.2022.1026593)

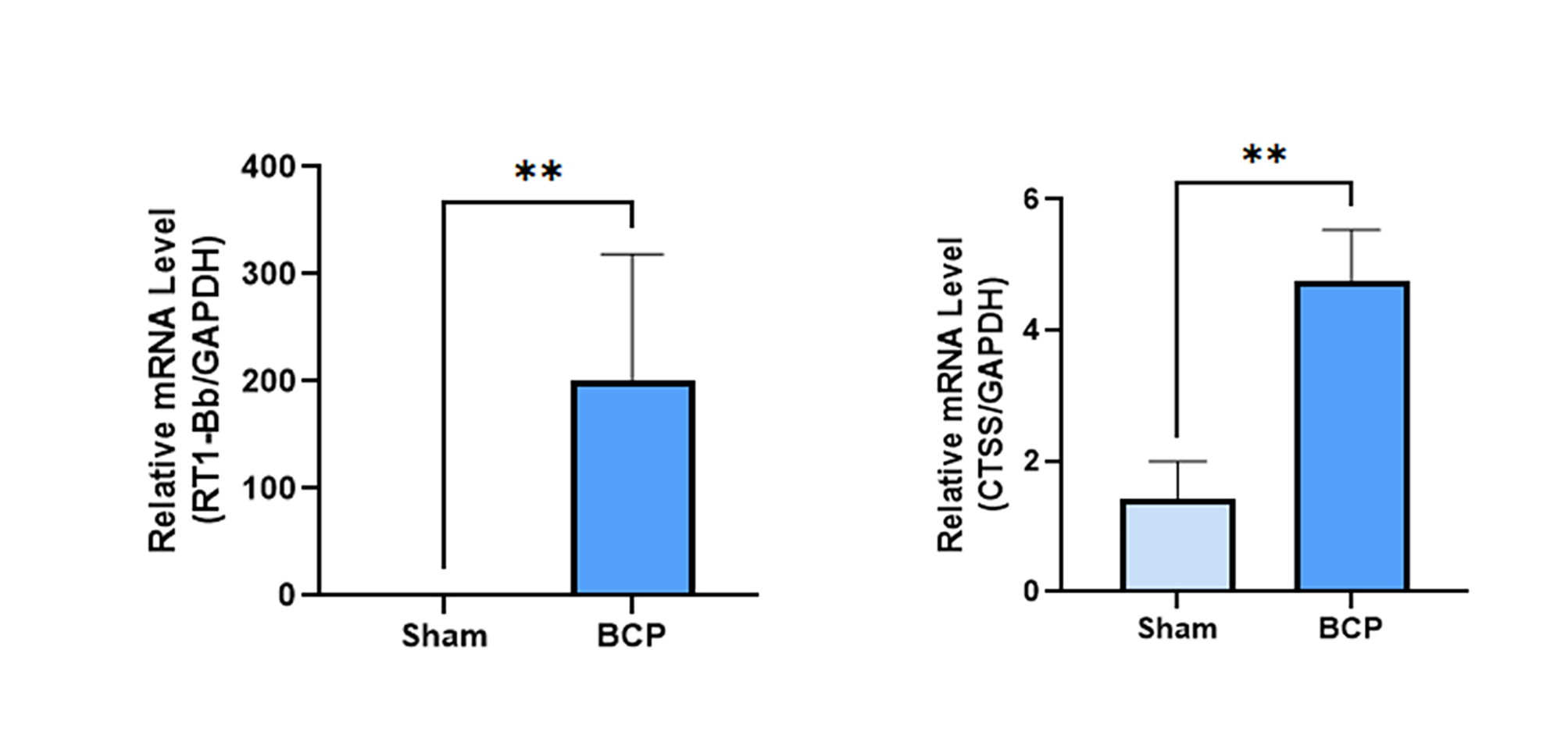

Supplement: Supplementary Figure 1 — The expression level of upregulated mRNAs (RT1-Bb and CTSS) in the mPFC of BCP rats. Data are expressed as mean ± SD and statistically analyzed by Student’s T-test. n = 5 per group. *p < 0.05, **p < 0.01 vs. Sham group. [file Image_1.tif]
